# Can Multiparametric Ultrasound Analysis Predict Malignancy in Testes? An 11-Year Single Center Experience with Testicular Masses

**DOI:** 10.3390/jcm13247853

**Published:** 2024-12-23

**Authors:** Yannic Volz, Isabel K. Brinkmann, Dirk-André Clevert, Nikolaos Pyrgidis, Patrick Keller, Philipp Weinhold, Friedrich Jokisch, Marc Kidess, Michael Chaloupka, Christian G. Stief, Julian Marcon

**Affiliations:** 1 Department of Urology, University Hospital, LMU Munich, 81377 Munich, Germanyjulian.marcon@med.uni-muenchen.de (J.M.); 2 Department of Radiology, University Hospital, LMU Munich, 81377 Munich, Germany

**Keywords:** testicular cancer, seminoma, ultrasound, contrast-enhanced ultrasound

## Abstract

**Introduction/Objective:** Contrast-enhanced ultrasound (CEUS) is a promising modality for differentiating benign and malignant lesions in various organs, including the testis. Testicular tumors, common in young men, are often treated with radical orchiectomy, which can have significant consequences. This study aimed to analyze CEUS parameters and their association with malignant testicular tumors. **Materials and Methods:** Between May 2009 and September 2020, 342 patients with suspected testicular lesions underwent CEUS at a tertiary referral center. Multiparametric ultrasound, including B-mode, Color Doppler, CEUS, and elastography, was performed. Exclusion criteria were the absence of a testicular lesion in the CEUS examination. Histological results, CEUS parameters, and elastography data were analyzed, and statistical correlations were evaluated using chi-square and Mann–Whitney U tests, with multivariate logistic regression for significant findings. **Results:** Of the 342 patients, 114 (33.3%) had suspicious CEUS findings, and 84 underwent surgical exploration. Malignancy was confirmed in 48 cases (57.1%). The sensitivity and specificity of CEUS for detecting malignancy were 93.8% and 85.2%, respectively. Contrast enhancement was observed in 93.3% of malignant tumors, but not statistically significant compared to benign lesions (*p* = 0.107). However, elastography revealed higher tissue stiffness in 77.8% of malignant tumors versus 41.0% in benign lesions (*p* = 0.009). Multivariate analysis indicated that tissue stiffness was significantly associated with malignancy (HR 8.29, 95%CI 1.26–54.58, *p* = 0.028). **Conclusions:** CEUS is a valuable tool for testicular lesion evaluation, with elastography showing strong potential in predicting malignancy through tissue stiffness. However, contrast enhancement and “wash-in/-out” were not reliable malignancy indicators. Further research is needed to standardize CEUS and elastography techniques for routine clinical use.

## 1. Introduction

With contrast-enhanced ultrasound (CEUS), a new imaging modality has arisen in recent years and has been used in the differentiation of benign and possibly malignant lesions in different organs, such as the liver or the kidney [1,2]. Recent evidence also suggests the usage of CEUS for the differentiation between pathologic and non-pathologic breast masses, leading to reduced costs and morbidity [3]. CEUS seems to provide a way of distinguishing malignant lesions from benign lesions, which might also be used for testicular lesions. Testicular tumors are amongst the most common malignant tumors in young adults and are classified into germ cell tumors, sex cord-stromal tumors, and others [4]. Most of these cases are germ cell tumors, which are further subclassified into seminomatous and non-seminomatous tumors [5]. In case of a suspicious testicular lesion, surgery with radical orchiectomy is commonly performed and may lead to infertility as well as great psychological and endocrine burden. Not uncommonly, orchiectomy specimens reveal non-neoplastic lesions, such as hematoma or necrosis, benign testicular tumors, such as Leydig cell tumors, and lymphoma, which do not require radical orchiectomy in retrospect. By distinguishing between malignant and benign lesions, CEUS might play a role in the diagnostic workflow for a testicular lesion. However, literature about this topic seems to be relatively scarce. This might be the reason why, so far, CEUS is not recommended in national and European guidelines for the diagnosis of testicular cancer [6,7]. Yet, previous results from recent literature look promising. A recent meta-analysis by Yu et al. found that CEUS can provide a better visualization of the vascularization of testicular lesion and, therefore, differentiate the type of testicular lesion accurately [8]. The accuracy of detecting and differentiating such lesions has been described with a sensitivity of up to 100% and a specificity of up to 93.8% [8,9]. Another group has also shown that the different parameters of CEUS allow for the characterization of the different lesions [10]. However, it is unclear which parameter of CEUS is related to an increased risk of being associated with a malignant lesion. Therefore, the aim of the current study is to analyze different parameters of the CEUS and their association with harboring a malignant tumor.

## 2. Material and Methods

The study was performed at our tertiary referral center as a single center. Between May 2009 and September 2020 all patients with a suspected malignant lesion underwent CEUS. Patients were usually referred to us by a urologist or general practitioner after suspicious testicular palpation or ultrasound. Oral and written informed consent of all patients were given before CEUS examination, and their associated risks and potential complications were carefully described. Exclusion criteria from the final analysis were the absence of a testicular lesion in the CEUS examination.

### 2.1. Multiparametric Ultrasound Examinations

All CEUS examinations were performed and analyzed by a single skilled radiologist with more than 15 years of clinical experience (EFSUMB Level 3). All included patients underwent native B mode, Color Doppler, CEUS scans, and strain elastography (SE) or shear wave elastography (SWE). Up-to-date, high-end ultrasound systems with adequate CEUS protocols were utilized (GE Healthcare: LOGIQ E9, GE Healthcare GmbH, Düsseldorf, Germany; Samsung RS80A Prestige, Samsungs Healthcare, Schwalbach, Gemany; Siemens Ultrasound Sequoia S2000, S3000, Siemens Healthcare Diagnostics GmbH, Vienna, Austria; Philips Ultrasound iU22, EPIQ 7, Philips GmbH Market DACH, Hamburg, Germany). A low mechanical index was used to avoid early destruction of microbubbles (<0.2). Only high-frequency ultrasound probes were used for the examinations. For all CEUS examinations, the second-generation blood pool contrast agent SonoVueVR (Bracco, Milan, Italy) was used. An amount of 2.4–4.8 mL of SonoVueVR was applied. After contrast agent was applied, a bolus of 5–10 mL sterile 0.9% sodium chloride solution was given. No adverse side-effects upon administration of SonoVueVR were registered. All CEUS examinations were successfully performed, and the image quality was sufficient in every single case, allowing for proper analysis of the sonomorphological appearance of the testicular lesions. The patient files and imaging records were retrieved from the picture archiving and communication system (PACS) of our institution.

**Figure 1:**
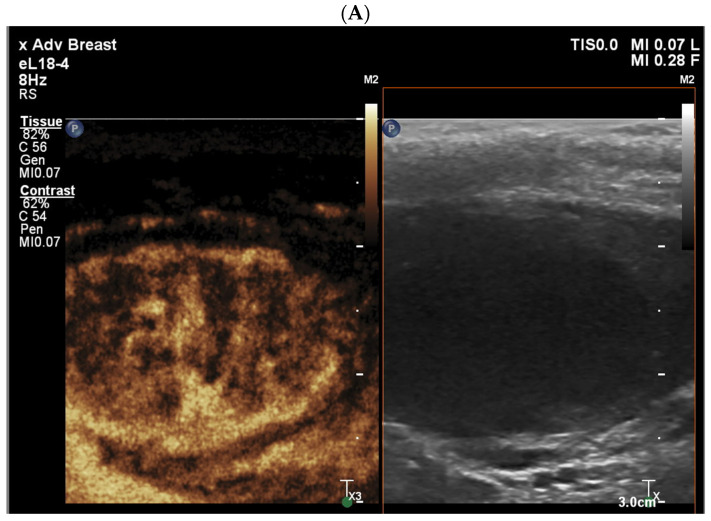

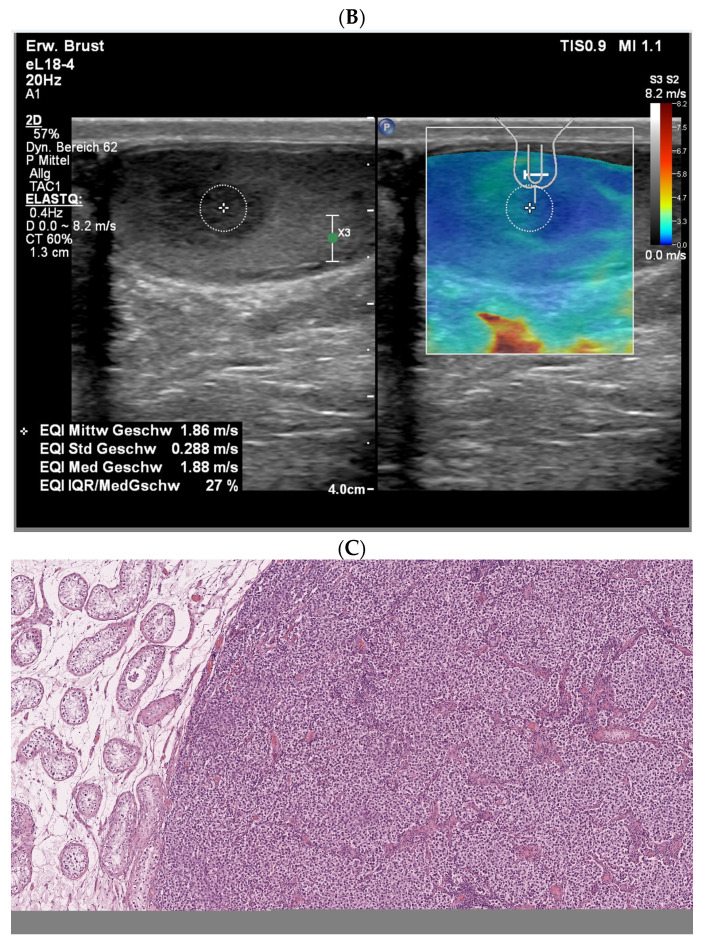
(**A**) Contrast enhancement in a patient with seminoma, (**B**) elastography in a patient with seminoma, and (**C**) histological slide of a patient with seminoma on the right and healthy testicular tissue on the left.

An evaluation of morphological features of the testicular tumors in native B mode included location, size, shape, and echogenicity of the tumors. Vascularization was evaluated using Color Doppler and CEUS (Figure 1A). “Wash-in” and “wash-out” were observed visually by the doctor performing the CEUS. Additional elastography evaluation, either by shear wave elastography (SWE) or strain elastography (SE), was performed in each case. For assessing tissue stiffness by SE, repeated compression and decompression of the testis by the probe was performed. In strain elastography, the stiffness of malignant and benign lesions and surrounding testicular parenchyma was visualized by color coding in real time. Stiffness was classified as ‘hard’, ‘soft’, or ‘equivalent’ compared to the surrounding parenchyma. In SWE, a 3 mm diameter region of interest (ROI) was placed in the longitudinal plane in native B mode into the testicular lesions and surrounding parenchyma. At least three shear wave velocity (SWV) measurements were performed in each examined case. Elastography is displayed in Figure 1B. Furthermore, the age of the patient as well as the final histological result were included. The examination was considered suspicious if a patient showed at least one feature positive (contrast enhancement including wash-in or wash-out of contrast agent and observation of stiffer areas during elastography).

### 2.2. Statistical Analysis

The correlation of CEUS variables and malignancy status was conducted with the chi-square test and Mann–Whitney U test. Only in the case of significant results in the univariate analysis was multivariate analysis performed by binary logistic regression. Normality was assessed using the Kolmogorov–Smirnov test. A *p*-value ≤0.05 was considered significant and reported two-sided. All statistical analyses were performed using SPSS (Version 26.0, IBM, New-York, NY, USA). This retrospective study was approved by the local ethical committee of the LMU University hospital with the approval number 20-1045. All study data were collected, according to the principles of the Declaration of Helsinki/Edinburgh 2002.

## 3. Results

### 3.1. General Patient Characteristics

Between 2009 and 2020 a total of 342 patients received CEUS of the testis as part of the diagnostic screening for testicular cancer. Of these, 114 (33.3%) patients showed a suspicious CEUS result. A total of 84 patients with suspicious results underwent surgical exploration, biopsy, or orchiectomy at our urology department. Thirty patients were lost to follow-up and may have undergone orchiectomy or further treatment at another department. Patients with a suspicious CEUS examination had a median age of 39 (IQR 33–47.5 years). Interestingly, as displayed in Table 1A, there was no statistical difference in the size of the testicular lesion between patients with a suspicious and non-suspicious CEUS examination (6.0 mm vs. 7.0 mm; *p* = 0.209). Four patients displayed malignant frozen section procedure results and immediately underwent radical orchiectomy. Regarding the final histological results, 32 patients (71.1%) revealed a seminoma of the testicle (Figure 1C), and 13 patients (28.9%) showed a non-seminomatous germ cell tumor. Looking at patients with malignant histology (Table 1B), we were not able to show statistically significant differences in age, lesion size germ cell tumor markers, or gonadotropin and testosterone levels (*p* > 0.05 in all cases).

### 3.2. Benign vs. Malignant Histology

The differences in CEUS parameters between patients with benign and malignant histology are summarized in Table 2. Contrast enhancement (Figure 1A) was observed in 93.3% of malignant tumors, but this did not occur at a statistically significantly higher rate compared to benign tumors (76.7%; *p* = 0.107). Similarly, rapid “wash-in” rates were not significantly different between malignant and benign lesions (68.8% vs. 64.1%; *p* = 0.250). Rapid “wash-out” was also present but without any statistically significant difference between the two groups. However, elastography revealed a marked distinction: 77.8% of patients with malignant tumors exhibited this feature, compared to only 41.0% of those with benign lesions. This difference was statistically significant (*p* = 0.009).

### 3.3. Positive CEUS Parameters and the Risk for a Malignant Lesion

Sensitivity was calculated at 93.8%; whereas, specificity was at 85.2%. Therefore, the positive predictive value was at 53.6% and the negative predictive value at 98.7%. Multivariable regression analysis of ultrasound parameters and their risk for a malignant lesion is displayed in Table 3. Higher tissue stiffness upon elastography showed a statistically significant impact in leading to the detection of a malignant lesion if positive (HR 8.29; 95%CI 1.26–54.58; *p* = 0.028). All other variables did not lead to a statistically significantly increased risk of a malignant lesion if positive in CEUS.

## 4. Discussion

Contrast-enhanced ultrasound (CEUS) is a new imaging modality, which can be used to differentiate benign and possibly malignant lesions in different organs, such as the liver or the kidney [1,2]. Previously, CEUS could provide the visualization of the vascularization of a testicular lesion and, consequently, differentiate the type of testicular lesion accurately [8]. With a sensitivity of up to 100% and a specificity of up to 93.8%, this new imaging modality sounds promising [8,9]. In our view, the validation of the detailed parameters to predict malignancy is needed. In the present study, CEUS was performed in 342 patients and yielded a suspicious result in 114 patients. Furthermore, 84 patients underwent surgical exploration at our department, and in 48 patients (57.1%), the final histopathological result revealed malignancy. In this study, we demonstrated that both the presence of contrast enhancement as well as a rapid “wash-in” and “wash-out” of contrast agent during the ultrasound examination were not associated with a malignant germ cell tumor of the testis. This finding is in line with the findings of Luzurier et al., who described a rapid “wash-in” in 69% of the malignant lesions as well as 73% of the benign lesions (*p* = 1.000) [11]. Still, other literature has shown that a rapid “wash-in” as well as “wash-out” was more often found in malignant lesions (77% vs. 25%, *p* < 0.001) [12], thus demonstrating that there is a broad heterogeneity of results. Rapid “wash-in” has even been associated with benign lesions, such as Leydig cell tumors [13]. This may be explained due to different CEUS systems used, as well as the different tumor architecture. Yet, it supports our findings that this parameter was not able to discriminate between benign and malignant lesions.

In our cohort, we found an overall sensitivity of 93.8% as well as a specificity of 85.2%. These results are well comparable to previous literature, where the overall sensitivity ranges between 73.0% [11,14], and the specificity range is even broader between 27.3% [15] and 96.0% [16]. Notably, elastography emerged as a significant predictor of malignancy, with increased tissue stiffness correlating strongly with malignancy. This finding corroborates results from Dikici et al., who demonstrated differential shear wave elastography (SWE) values between normal testicular parenchyma (4.4 ± 3.9 kPa), seminomas (10.6 ± 3.9 kPa), and non-seminomatous germ cell tumors (47.0 ± 14.5 kPa) [17]. Similar results have been presented for strain elastography. Aigner et al. conducted a qualitative analysis using strain elastography (SE) and found that all neoplasms in their patient cohort (n = 50) appeared as blue (indicating hardness) on the colorimetric map. Additionally, three non-neoplastic lesions (a post-biopsy scar, a cyst, and a partial infarction) were also detected as hard, while other non-neoplastic lesions were labeled as soft. In their study, SE achieved 100% sensitivity, 81% specificity, a 100% negative predictive value, 92% positive predictive value, and 94% accuracy in diagnosing testicular tumors [18]. Konstantatou et al. arrived at different conclusions in their research [19]. While they observed a strong correlation between strain ratio and qualitative analysis, a comparison of ROC curves revealed that the strain ratio was more effective in diagnosing malignancies. However, they found no significant difference between the two methods when it came to identifying neoplastic lesions. These heterogenous results may be explained by the fact that the pressure applied during elastography is dependent on the examiner, and this is not taken into account in the calculation of tissue stiffness [20]. In another study by Marcon et al., it was observed that focal testicular lesions are stiffer compared to normal parenchyma, as measured by both SWE and virtual touch imaging quantification (VTIQ). The study also noted that VTIQ produced higher stiffness values than SWE, likely due to the technical differences inherent to each method [21]. Still, after multivariate analysis hard elastography remained as the only significant predictive factor for malignancy in the lesion and can, therefore, serve as a surrogate parameter. The sensitivity has been described to be as high as 87.5% with a specificity of 98.2% [22]. Also, for other tumor, entities like breast cancer elastography have been proven to differentiate between benign and malignant lesions [23].

Despite these promising findings, the routine clinical use of multiparametric ultrasound, including CEUS and elastography, presents challenges. Additionally, more staff members are needed, all of whom must be adequately trained. For contrast-enhanced ultrasound (CEUS), for instance, at least two operators are necessary—one to perform the scan and another to administer the contrast medium. Training is especially important for strain elastography (SE), as it is highly operator-dependent and requires mastery of proper free-hand compression techniques. Additionally, the compression and decompression of the testis may cause mild discomfort for the patient; however, it was not perceived as painful. Another challenge is the higher cost associated with multiparametric ultrasound compared to standard ultrasound, considering the need for additional staff, the expense of contrast agents, and the investment in specialized software and hardware for CEUS and elastography. Moreover, with elastography, there are notable inconsistencies in results across different equipment brands, making standardization difficult [24,25]. Overall, most of our patients showed testicular lesions smaller than one centimeter and, therefore, represent a cohort with unmet needs. Orchiectomy is the current practice in most of these patients. CEUS seems to provide a tool, which may be offered as surveillance in small testicular masses, thus maybe even reducing the need for unnecessary orchiectomy or surgical exploration.

The present study is not without limitations. First, the study was performed by retrospectively assessing available patient information, which may lead to missing data. In line with that, we are not able to provide whether patients were referred due to a palpable lump or a suspicious routine ultrasound by their urologist or general practitioner. Furthermore, absolute numbers for SWE and SE are missing; for example, data were only extracted from the written reports, where the elastography was either determined as hard or soft. A similar problem accounts for absolute numbers of “wash-in” or “wash-out” time. Moreover, only 84 patients underwent testicular surgery of the 114 patients that had a suspicious CEUS finding. The 30 missing patients may have undergone surgery at another institution leading to the limitation that we do not possess information about their histological result. Furthermore, different ultrasound devices were used for the examinations with different signal behavior, therefore limiting the comparability of results.

## 5. Conclusions

In conclusion, this study demonstrates that contrast-enhanced ultrasound (CEUS) is a promising tool for the evaluation of testicular lesions, but not all parameters consistently predict malignancy. While contrast enhancement and rapid “wash-in”/“wash-out” were not reliable indicators of malignancy, elastography seems to be significantly associated with malignant tumors. Specifically, higher tissue stiffness correlated with a higher risk of malignancy. Despite these findings, the application of multiparametric ultrasound, including CEUS and elastography, faces challenges, such as operator dependency, equipment variability, and increased costs. Further research is needed to standardize these techniques and validate their clinical utility in testicular cancer diagnostics.

## Figures and Tables

**Table 1:** (**A**) General characteristics of all patients with CEUS (excluding the 30 patients that were lost to follow up). (**B**) General characteristics of patients undergoing surgical exploration.

**(A) General characteristics of all patients with testicular CEUS**				
Variable	**Total (n = 312)**	**Ultrasound suspicious (n = 84)**	**Ultrasound non-suspicious (n = 228)**	***p-value***
Age, years	38.5 (32–46)	39 (33–47.5)	42 (32.5–57)	0.491
Lesion size, mm	7 (4–12)	6.0 (3.9–10.3)	7.0 (2.2–10.0)	0.209
Malignant histology	48 (15.4%)	45 (53.6%)	3 (1.3%)	<0.001
**(B) General characteristics of all patients undergoing surgical exploration**				
	**Total (n = 84)**	**Malignant histology (n = 48)**	**Benign histology (n = 39)**	***p-value***
Age, years		39 (32–46)	39 (32–46)	0.750
Lesion size, mm		8.0 (4.0–12.0)	5.0 (3.3–10.5)	0.273
LDH serum level		204.0 (176.5–238.5)	202.0 (178.0–243.0)	0.775
AFP serum level		2.9 (2.1–4.2)	2.7 (1.6–3.9)	0.432
betaHCG serum level		1.0 (1.0–1.5)	1.0 (1.0–1.0)	0.101
Testosterone serum level		4.5 (1.9–5.6)	3.5 (1.8–4.3)	0.631
LH serum level		6.9 (6.3–18.7)	8.3 (4.8–12.3)	0.742
FSH serum level		10.7 (2.4–23.8)	24.3 (4.2–30.3)	0.470

**Table 2:** Differences in CEUS parameters in patients with benign and malignant histology.

Variable	Malignant Histology	Benign Histology	*p*-Value
Contrast enhancement	42 (93.3%)	30 (76.7%)	0.107
Rapid “wash-in”	31 (68.8%)	25 (64.1%)	0.250
Rapid “wash-out”	2 (0.04%)	2 (0.05%)	0.334
Elastography hard	35 (77.8%)	16 (41.0%)	**0.009**

**Table 3:** Multivariate analysis for predictors for malignant histology.

Variable	HR	95% Confidence Intervall	*p*-Value
Rapid “wash-in”	0.0	0.0–0.0	0.999
Elastography soft	6.11	0.17–215.60	0.320
Elastography hard	8.29	1.26–54.58	**0.028**
Hypoechogenic	0.26	0.04–1.75	0.166
Hyperechogenic	0.13	0.01–3.66	0.229

## Data Availability

The datasets presented in this article are not readily available due to ethical/legal obligations. Requests to access the datasets should be directed to corresponding author.
